# Short-term enhancement of cognitive functions and music: A three-channel model

**DOI:** 10.1038/s41598-018-33618-1

**Published:** 2018-10-19

**Authors:** Ashish Gupta, Braj Bhushan, Laxmidhar Behera

**Affiliations:** 1 Department of Electrical Engineering, Indian Institute of Technology Kanpur, Kanpur, 208016 India; 2 Department of Humanities and Social Sciences, Indian Institute of Technology Kanpur, Kanpur, 208016 India

## Abstract

Short-term effects of music stimulus on enhancement of cognitive functions in human brain are documented, however the underlying neural mechanisms in these cognitive effects are not well investigated. In this study, we have attempted to decipher the mechanisms involved in alterations of neural networks that lead to enhanced cognitive effects post-exposure to music. We have investigated the changes in Electroencephalography (EEG) power and functional connectivity of alpha band in resting state of the brain after exposure to Indian classical music. We have quantified the changes in functional connectivity by phase coherence, phase delay, and phase slope index analyses. Spatial mapping of functional connectivity dynamics thus obtained, on brain networks revealed reduced information flow in long-distance connections between frontal and parietal cortex, and between other cortical regions underpinning intelligence. Analyses also showed increased power in the prefrontal and occipital cortex. With these findings, we have developed a stimulus-mechanism-end effect based neuro-cognitive model that explains the music induced cognitive enhancement by a three-channel framework - (1) enhanced global efficiency of brain, (2) enhanced local neural efficiency at the prefrontal lobe, and (3) increased sustained attention. Results signify that music directly affects the cognitive system and leads to improved brain efficiency through well-defined mechanisms.

## Introduction

Music has been identified as a useful stimulus in various healing and rehabilitation practices^1–3^. Earlier studies showed that a short time exposure to music can also enhance the spatio-temporal performance of brain regions^4,5^, popularly known as the Mozart effect. However, subsequent findings claimed that the effect is not limited to Mozart’s composition^6,7^ or to spatio-temporal reasoning^8,9^. Further studies proposed that any stimulus that induces a moderate level of arousal and pleasant mood in the subject results in a significant enhancement in the cognitive performances^6,10,11^ and that this effect disappears when arousal and pleasantness are held constant^12^. This led to arousal-mood hypothesis which states that the enhanced cognitive performances are effects of stimulus on the mood of the subject; there is no music induced direct activation of neurons to enhance cognitive performances^11^. A comprehensive review by Schellenberg *et al*.^8^ concluded that music listening improves cognitive performance, albeit only in the short term. Results from a recent review, however, emphasized that music listening has many inherent benefits for cognitive, motor, emotional, and social functioning^13^. On the other hand, few earlier studies supported the claim that music directly influences brain networks for enhancing spatial abilities^4,14,15^. Other studies showed that music activates brain regions responsible for attention and cognitive tasks^16–18^. A recent finding suggests that music activates regions which are linked to memory, cognition, and IQ^19^. However, the previous studies were not able to convincingly establish the detailed and systematically parameterize direct mechanisms between music and cognitive enhancement. Few recent works reveal a new aspect of interconnections between music and brain signals^20^. Music and signals such as EEG and fMRI share a striking similarity between them, of being scale free in nature^20,21^. Indeed, sonification of brain signals into music has given a deeper insight into neuro-activities of the brain from musical perspective^21^.

The apparent difference between the findings of the previous studies can be partly attributed to the lack of in-depth knowledge of mechanisms involved in these stimulus-end effect based works^8,19^. To the best of our knowledge, detailed study of brain dynamics on exposure to music with respect to spectral power and functional connectivity of cortical regions of brain has not yet been done. Hence, a well-defined quantitative framework is required to clearly investigate the connections between music and cognitive effects among individuals. In this work, we hypothesized that music, as an external stimulus, directly induce alterations in brain network dynamics to create short-term cognitive enhancement. To understand these mechanisms, we selected two important neuropsychological constructs – intelligence and sustained attention.

Numerous groups have researched on Intelligence Quotient (IQ) to account for the observed variation in cognitive performances between individuals. Two complementary theories have been well received in this regard— the Neural efficiency hypothesis of Intelligence (NEH), and the Parieto-Frontal Integration Theory of intelligence (P-FIT). NEH postulates that individuals with higher IQ perform a cognitive task with fewer brain resources compared to their counterparts^22^. P-FIT theory proposes that the frontal and parietal parts of the brain are centres primarily responsible for intelligence, along with the cingulate cortex, temporal lobe, occipital lobe and association cortices between frontal and parietal lobes^23^. Accordingly, a number of EEG variables have been shown to strongly correlate with the IQ of the individual. EEG power and coherence are positively and negatively correlated with IQ respectively^24^. Enhanced power is observed in high IQ participants in the frontal cortex, in particular in the prefrontal cortex (area linked to cognitive functioning) and in the occipital cortex^22,24–27^. Connectivity measures such as phase delay for short inter-electrode distance especially in the frontal lobe, have been found to reduce in high IQ individuals suggesting speedy processing at the frontal cortex^24^. Phase slope index for long inter-electrode distance has been found to reduce in participants with high IQ^28^ especially between frontal and parietal lobe implying reduced information flow between them; optimization of the widespread activity of the brain resources for efficient functioning. This led to small world characteristics in high IQ subjects with increased hub order at the frontal and parietal lobes^28,29^. Recent fMRI studies have also led to a resurgence in the importance of brain areas related to temporal, occipital, and insular cortex in cognitive processing^30,31^.

Sustained attention is vital for any task performance. It serves prominently three purposes – (a) excitation of task-relevant processes, (b) monitoring and evaluating of ongoing cognitive processes, and (c) inhibition of task-irrelevant processes^32^. The important regions responsible for sustained attention is fronto-parietal system^33^. Some preliminary studies have indicated that music listening broadens the range of attention^34^ and a notable contribution to intelligence’ variance is explained by attention control capacities^35–37^. Thus, this suggests a possible link among music, attention, and intelligence.

Multiple types of oscillatory signals – theta, delta, alpha, beta, and gamma signals, modulate brain functions at all sensory and cognitive levels. Hence, selection of the most suitable oscillation band fundamental to cognitive processes is vital. Alpha band oscillations are known to play an active role in cognitive process^38–40^. Furthermore, the most significant correlations between music and the psychometric measures of IQ have been consistently found in the alpha band^18,22,25–28,41^. Hence, in the present work, we focused our analyses on the alpha band spectrum.

Another important aspect in establishing a quantitative framework of mechanisms in music induced cognitive changes is the type of music that is used in the experimental study. Earlier studies used mostly Western music, especially Mozart, leaving scope to question whether other music forms from different cultural backgrounds would also show similar neural mechanisms. We used a famous eastern instrumental composition of Raga Darbari, played on flute by a professional Indian musician, as the stimulus in the present study. The stimulus maintains the cultural salience of the music for the participants.

In summary, with Raga Darbari as the external musical stimulus, we investigate the changes in EEG patterns of brain networks during the resting state with the intent to explore the neural mechanisms responsible for enhanced cognitive abilities. Primarily, we tested (a) changes in alpha power at the prefrontal and occipital cortex, and (b) variations in the information flow between long-distance connections in alpha band, especially between frontal and parietal cortex. Based on findings from these experiments, we propose a novel comprehensive model to explain the role of music in stimulating the dedicated regions of brain that lead to observed cognitive effects.

## Results

### Phase coherence analysis

**Figure 1:**
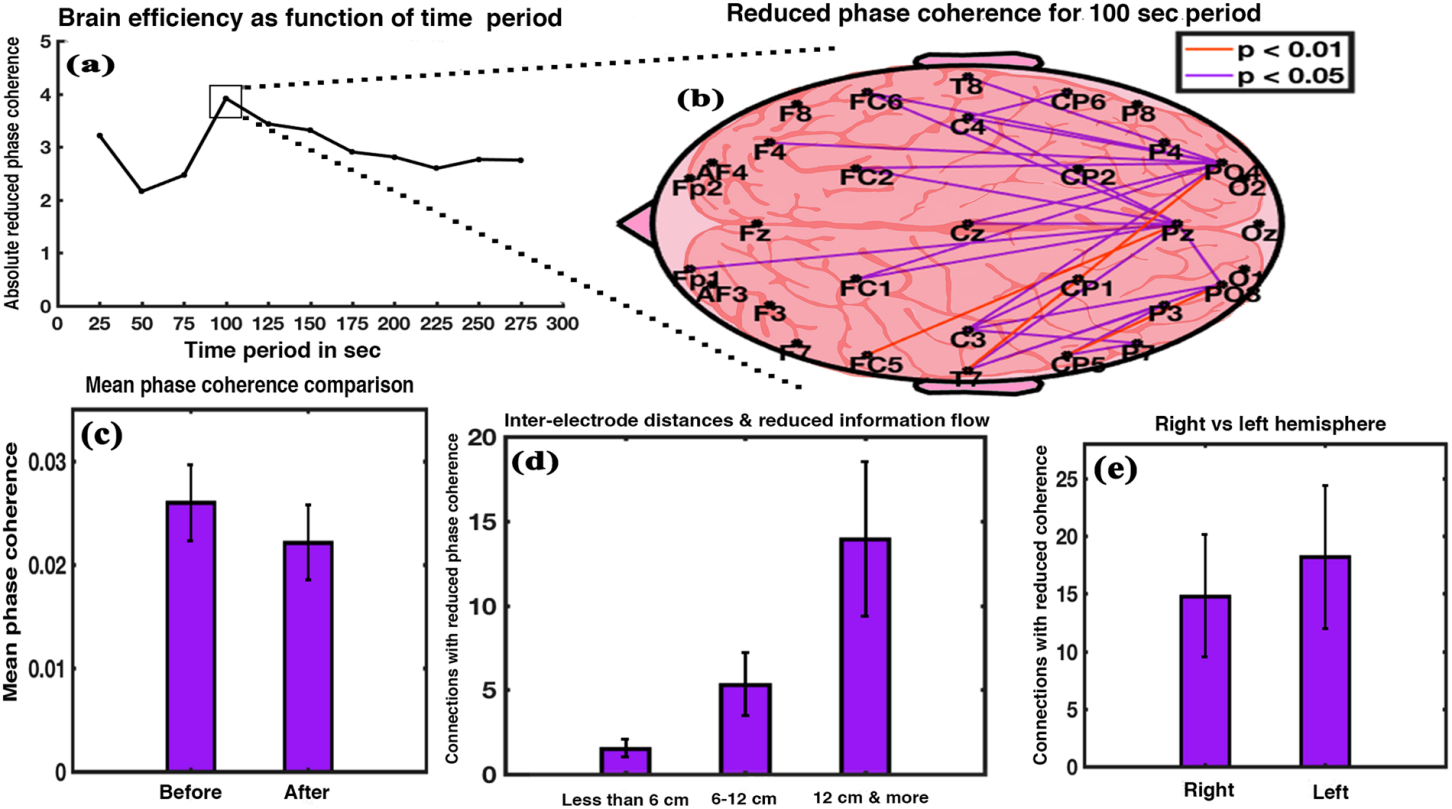
Phase coherence analysis. (**a**) Cumulative reduced phase coherence of the interconnections with significant reduction in coherence after music exposure as a function of time period. (**b**) Interconnections (27) between the electrodes with significant reduction in the coherence value for 100 sec period. (**c**) Relative mean coherence of the 27 connections showing significant difference between before and after music stimulus. (**d**) Number of connections with significantly reduced coherence as function of inter-electrode distances. (**e**) Hemispheric difference after exposure to music with respect to number of connections having significantly reduced coherence after music stimulus (error bars = 1 SD).

There was no significant increment of the phase coherence between any electrodes between pre-music silence and post-music silence condition except a few interconnections. Nevertheless, several connections experienced a significant reduction in coherence value after exposure to music. Cumulative reduced phase coherence (sum of reduced coherence values of connections for which a significant reduction occured on exposure to music) after music stimulus as a function of the time period is shown in Fig. 1(a). We observed a significant reduction in phase coherence post music for all the time periods with a peak at 100 seconds. Brain efficiency is known to be inversely proportional to phase coherence^24^. Thus, the findings imply that the brain remained efficient for the entire time duration with a peak at 100 seconds’ time period. We then analysed the brain state for the duration of first 100 seconds to study the maximum effect of music listening. Figure 1(b) shows the brain network inter-connections for 100 seconds’ time period, which showed a significant reduction (Wilkinson sign rank test, p < 0.05) in coherence on exposure to music with Z value ranging between 1.9879 and 2.7830 and effect size between 0.3629 and 0.5081. We found connections (27 in total) interlinking parietal and occipital brain regions with frontal, central, and temporal brain regions as well as interlinking parietal with occipital brain region (Supplementary Fig. S1). Figure 1(c) shows comparison between the mean values of the 27 connections. It depicts an effective reduction in brain network’s coherence (thus information flow) at 100 seconds, signifying net enhancement in brain efficiency. It shows a significant reduction after music stimulus (t = 4.3304, df = 14, p < 0.001, effect size = 1.1181). Figure 1(d) shows the number of connections with reduced coherence as a function of inter-electrode distance. A repeated measure ANOVA with a Greenhouse-Geisser correction indicates a significant effect of inter-electrode distance on the number of connections (F_1.270,17.777_ = 100.980, p < 0.001). Post hoc comparisons using Bonferroni correction show that connections in the range of 6–12 cm (Mean = 5.33, SD = 1.877) were greater in number than those in the range of less than 6 cm (Mean = 1.53, SD = 0.516) and the difference was statistically significant (p < 0.001). Connections in the range of more than 12 cm (Mean = 13.93, SD = 4.574) were significantly greater than those in the range of 6–12 cm (p < 0.001) and also greater than those in the range of less than 6 cm (p < 0.001). Thus, long-distance connections were affected more than the short-distance ones. Figure 1(e) shows that the left hemisphere of the brain had more number of connections with reduced coherence as compared to the right hemisphere (t = −2.3716, df = 14, p < 0.05, effect size = −0.6124). The results show reduced communication post music.

### Phase delay analysis

**Figure 2:**
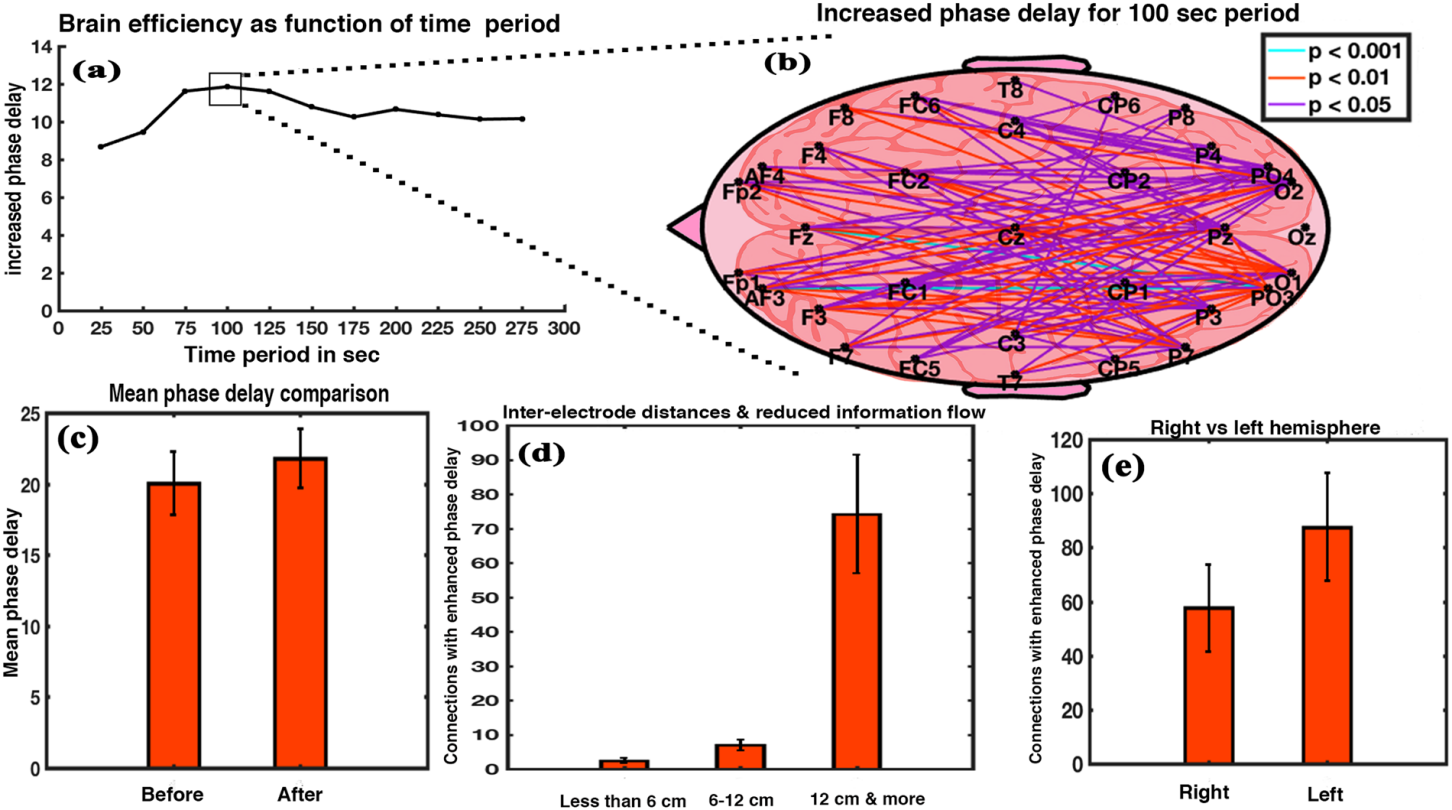
Phase delay analysis. (**a**) Cumulative increased phase delay of the interconnections with significant increment in phase delay after music exposure as a function of time period. (**b**) Interconnections (104) between the electrodes with significant increment in the phase delay value for the 100 sec period. (**c**) Relative mean value of phase delay of the 104 connections showing significant difference between before and after music stimulus. (**d**) Number of connections with significantly enhanced phase delay as a function of inter-electrode distances. (**e**) Hemispheric difference after exposure to music with respect to number of connections having significantly enhanced phase delay after music (error bar = 1 SD).

Figure 2(a) shows the variation in the cumulative increment of phase delay as a function of the time period. Phase delay is inversely related to information flow and thus directly proportional to brain efficiency^24^. Cumulative phase delay continuously increased till 100 seconds confirming the period of 100 seconds as the most efficient period. After that, the efficiency of the brain stayed moderately enhanced. Brain network connections for the period of 100 seconds are shown in Fig. 2(b). A total of 104 connections showed significant increment (Wilkinson sign rank test, p < 0.05) in phase delay with Z value ranging between −1.9879 and −3.2942 and effect size between −0.3629 and −0.6014. We found connections interlinking parietal and occipital brain regions with frontal, central, and temporal brain regions as well as interlinking parietal with occipital brain region (Supplementary Fig. S1). Figure 2(c) demonstrates mean phase delay for the 104 connections. It depicts an effective increment in brain network’s phase delay at 100 seconds, signifying net enhancement in brain efficiency. Mean phase delay increased after exposure to music (t = −4.8745, df = 14, p < 0.001, effect size = −1.2586). Variation in the number of connections with increased phase delay as a function of inter-electrode distance is shown in Fig. 2(d). A repeated measure ANOVA with a Greenhouse-Geisser correction indicates a significant effect of inter-electrode distance on the number of connections (F_1.010,14.140_ = 259.504, p < 0.001). Post hoc comparisons using Bonferroni correction showed that connections in the range of 6–12 cm (Mean = 7.000, SD = 1.60357) were greater in number than those in the range of less than 6 cm (Mean = 2.4667, SD = 0.74322) and the difference was statistically significant (p < 0.001). Connections in the range of more than 12 cm (Mean = 74.2000, SD = 17.25109) were significantly greater than those in the range of 6–12 cm (p < 0.001) and also greater than those in the range of less than 6 cm (p < 0.001). Thus, the long-distance connections were affected more than the short-distance ones. With respect to the hemispheric differences, left hemisphere of the brain had more number of connections with enhanced phase delay (Fig. 2e) as compared to the right hemisphere (t = −6.8362, df = 14, p < 0.001, effect size = −1.7651). The result shows a reduction in the network communication post music.

### Phase Slope Index analysis

**Figure 3:**
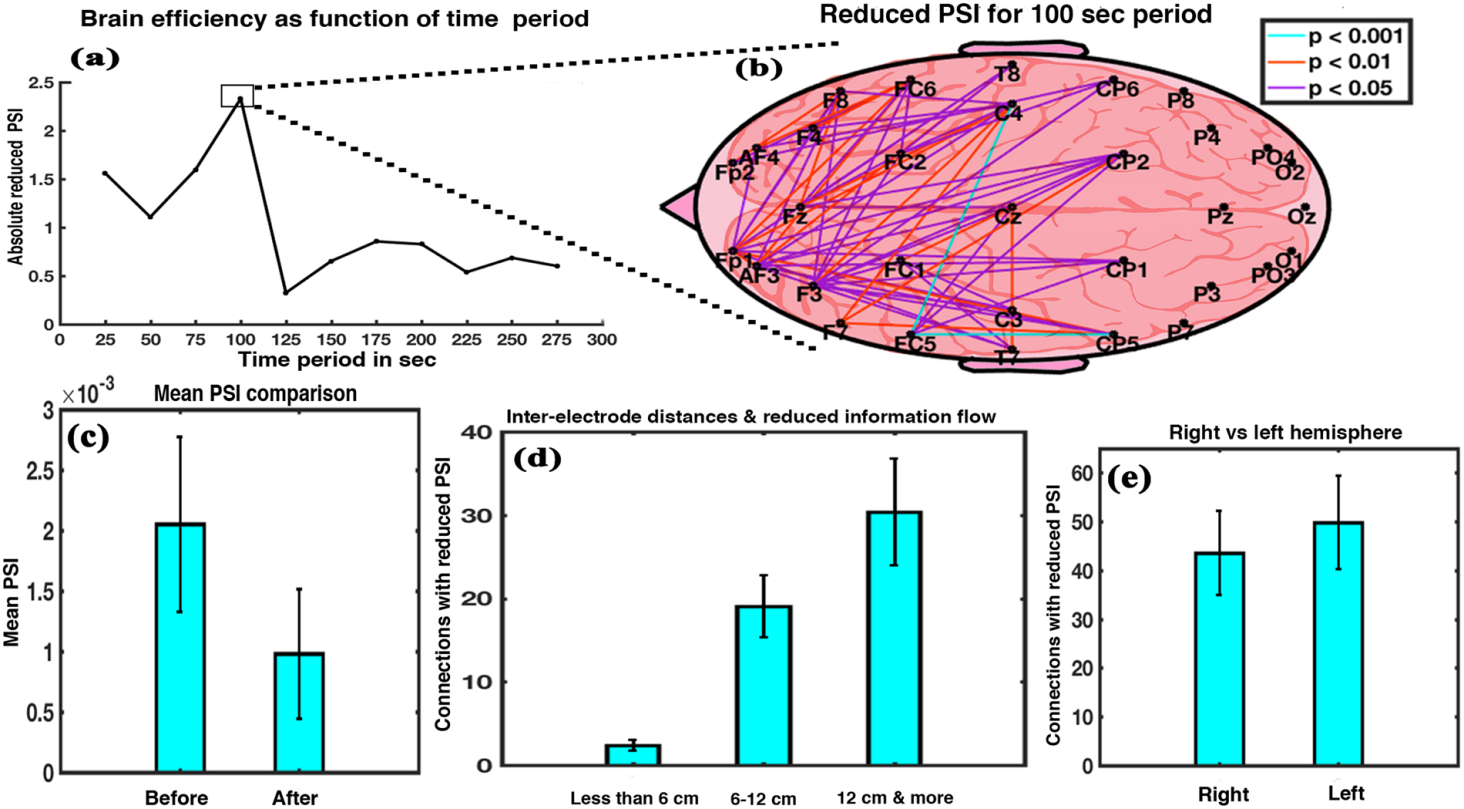
Phase slope index analysis. (**a**) Cumulative reduced phase slope index (PSI) of the interconnections with significant reduction in PSI after music exposure as a function of time period. (**b**) Interconnections (66) between the electrodes with significant reduction in the PSI value for the 100 sec period. (**c**) Relative mean PSI of the 66 connections showing significant difference between before and after music stimulus. (**d**) Number of connections with significantly reduced PSI as a function of inter-electrode distances. (**e**) Shows hemispheric difference after exposure to music with respect to number of connections having significantly reduced PSI after music (error bars = 1 SD).

Figure 3(a) depicts the cumulative reduction in the PSI as a function of the time period on music listening. A significant reduction in PSI for all the time periods was obtained, though it was most prominent for 100 seconds’ period. Reduced PSI value is an index of the Brain efficiency^28^, and hence the finding reconfirms that the brain was most efficient during 100 seconds’ period. Figure 3(b) shows brain network connections after 100 seconds for which there was a significant reduction in PSI on exposure to music. A total of 66 connections, interlinking frontal region with central, parietal, and temporal regions as well as interlinking intra-frontal region (Supplementary Fig. S1), were found to have significantly reduced PSI value after listening to music (Wilkinson sign rank test, p < 0.05). Z value ranged between 1.9879 and 3.1238 and effect size ranged between 0.3629 and 0.5703. The PSI value of only one connection significantly increased after exposure to music. Figure 3(c) shows the mean values of the 66 connections which significantly reduced in the post-music condition (t = 5.9161, df = 14, p < 0.001, effect size = 1.5275). It depicts an effective reduction in brain network’s PSI value at 100 seconds, signifying net enhancement in brain efficiency. Figure 3(d) shows the number of connections with reduced PSI as a function of inter-electrode distance. A repeated measure ANOVA with a Greenhouse-Geisser correction indicates a significant effect of inter-electrode distance on the number of connections (F_1.407,19.699_ = 254.874, p < 0.001). Post hoc comparisons using Bonferroni correction show that connections in the range of 6–12 cm (Mean = 19.07, SD = 3.731) were greater in number than those in the range of less than 6 cm (Mean = 2.40, SD = 0.632) and the difference was statistically significant (p < 0.001). Connections in the range of more than 12 cm (Mean = 30.400, SD = 6.423) were significantly greater than those in the range of 6–12 cm (p < 0.001) and also greater than those in the range of less than 6 cm (p < 0.001). Thus, the long-distance connections were affected more than the short-distance ones. Findings also indicate that the left hemisphere of the brain had more number of connections (Fig. 3e) with reduced PSI as compared to the right hemisphere (t = −4.2577, df = 14, p < 0.001, effect size = −1.0993). The result shows a reduction in information flow post music.

### Power analysis

**Figure 4:**
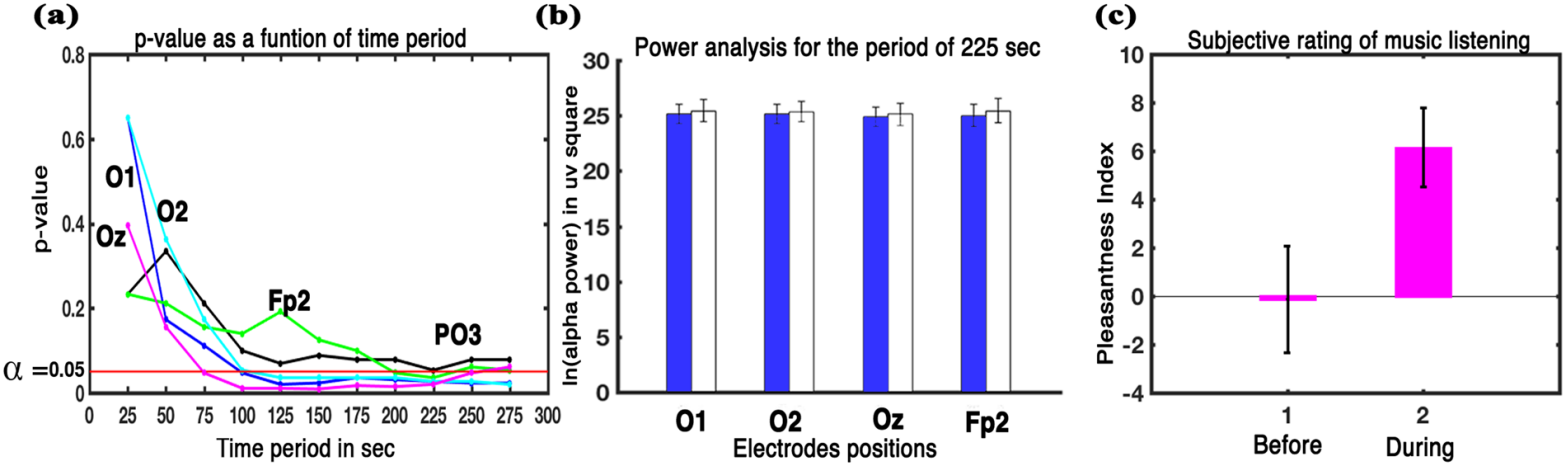
Power analysis. (**a**) Depicts p-value for the increment in the power of post music silence condition as a function of time period. Fp2 showed significant increment only for 200 sec and 225 sec time period while occipital lobe showed enhanced increment for any time duration more than 125 sec, (**b**) Relative log transformed alpha power value before and after music stimulus for O1, O2, Oz and Fp2 electrodes positions for the 225 sec period that showed significant enhancement (error bars = 1 SD), (**c**) Illustrates the mean subjective assessment of mood before and after listening the music. Music was successful in inducing a moderate level of pleasantness in the participants (errorbars = 1 SD).

We examined alpha power variation in the prefrontal and occipital lobes which are correlated with general Intelligence^24,26^. Figure 4(a) illustrates the p-value for prefrontal, occipital, and parietal electrodes corresponding to the hypothesis that there is no difference between pre-music and post-music condition in power value. Wilkinson sign rank test was used for the power analysis comparison. Significantly enhanced alpha power at the Fp2 electrode position was found for the time period of 200 seconds (Z = −1.9879, p < 0.05, effect size = −0.3629) and 225 seconds (Z = −2.1015, p < 0.05, effect size = −0.3837) indicating a late rise for a brief period in alpha power at the prefrontal cortex. Occipital cortex particularly showed enhanced power. All the three sites in the occipital cortex, O1, O2, and especially Oz, showed significant increment for the 125 seconds’ time period onwards and remained enhanced significantly for most of the time. Electrode O1 showed a maximum enhancement at the time period of 125 seconds (Z = −2.3286, p < 0.05, effect size = −0.4251), O2 at 275 seconds’ period (Z = −2.3286, p < 0.05, effect size = −0.4251), while Oz at 150 seconds’ period (Z = −2.6126, p < 0.01, effect size = −0.4770). Parietal electrode PO3 showed a feeble increment in power at 225 seconds’ period although not significant (Z = −1.9311, p = 0.0535, effect size = −0.3526). Thus, the finding supports increment in cognitive performance. Figure 4(b) shows the log-transformed alpha power for the brain state before and after music listening under the resting condition for the 225 seconds’ duration for which a significant enhancement was obtained at O1 (Z = −2.2151, p < 0.05, effect size = −0.4044), O2 (Z = −2.2151, p < 0.05, effect size = −0.4044), Oz (Z = −2.3286, p < 0.05, effect size = −0.4251), and Fp2 (Z = −2.1015, p < 0.05, effect size = −0.3837) electrode’s sites.

### Subjective assessment of listening to music

The participants expressed whether the music was pleasant or unpleasant. They then rated their mood before and after listening to the music on an 11-point Likert scale where 1 = least, 10 = most, and 0 represents the neutral value. The mean subjective rating before listening to the music (Mean = −0.1333, SD = 2.1996) increased (Mean = 6.1333, SD = 1.6417) showing significant enhancement (t = −9.5232, df = 14, p < 0.001, effect size = −2.4589) on listening to the music (Fig. 4c). The result indicates a moderate level of pleasant experience after listening to the music.

## Discussion

Music is linked to short-term enhancement of cognitive processing^4,5^. The process is mediated through arousal and mood of the subject as induced by the music^10,11^. However, due to the lack of clear and direct mechanisms demonstrating the cognitive effect of music on the brain, the end effect of music has been questioned in several studies^6,12^. The present study explores the neural mechanisms accountable for enhanced intelligence on exposure to music. We studied the changes in the characteristics of the brain networks during the resting state of the brain upon listening to music. Primarily we looked into the alteration in the functional connectivity and EEG power of the brain network in the alpha band.

Based on earlier works^24,28,42,43^ we applied three phase analyses measures to comparatively study functional connectivity of brain networks from different perspective; namely phase coherence, phase delay, and PSI. Phase coherence strictly identifies phase synchronized interconnections of brain networks independent of amplitude^42,43^. Phase delay analysis measures mean phase angle between two-time series^24^. Phase slope index, on the other hand, measures effective connectivity of the brain networks. It measures frequency average of the slope of phase of coherence^28^. The analysis of PSI involves only imaginary part, thereby making it robust to volume conduction. All the three metrics measure different features of the brain networks while quantifying associated information flow. Analysing the EEG data from different perspective would help in a robust characterization of the functional connectivity of the brain.

Study conducted by Thatcher *et al*.^24^ reported that biomarkers based on phase information are more strongly correlated to IQ than those based on power. Thus, our first probing method, phase coherence analysis is quite suitable. Findings show a significant diminution in mean phase coherence of the brain (Fig. 1c), and therefore net reduction in the information flow between various cortical regions upon listening to music. This signifies net enhancement in brain efficiency^24^. The effect size obtained for phase coherence analysis was 1.1181. We found that connections (27 in total) with reduced coherence were interlinking parietal and occipital brain regions with frontal, central, and temporal brain regions as well as interlinking parietal with occipital brain region.

Phase delay biomarker, our next probing technique, had been shown to have the strongest correlation, the largest number of inter-connections significantly correlating with respect to IQ among other biomarkers such as spectral coherence, absolute power, etc^24^. Phase delay is inversely related to information flow between two cortical regions. Results showed a significant enhancement of mean phase delay value of the brain (Fig. 2c) thereby, a reduction in the information flow between various cortical regions of the brain after listening to music. This signifies net enhancement in brain efficiency^24^. We found that connections (104 in total) with enhanced phase delay were interlinking parietal and occipital brain regions with frontal, central, and temporal brain regions as well as interlinking parietal with occipital brain region. The absolute value of effect size obtained for phase delay was 1.2586.

Phase slope index, which is directly proportional to information flow between brain cortical regions, measures the functional connectivity between those regions but also accounts for the variation in power along with phase of the respective regions. Results showed a significant reduction of mean PSI value of the brain (Fig. 3c) thereby reconfirming reduction in information flow post music between cortical regions of the brain. This signifies net enhancement in brain efficiency^28^. The effect size obtained for PSI analysis was found to be 1.5275. We found that connections (66 in total) with reduced PSI were interlinking frontal region with central, parietal, and temporal regions as well as interlinking intra-frontal region.

These results indicate a reduction in the information flow between various cortical regions of the brain on exposure to music. Decreased information flow has been linked to high IQ and implies a more efficient brain^24,28^.These results are in line with the NEH theory.

**Figure 5:**
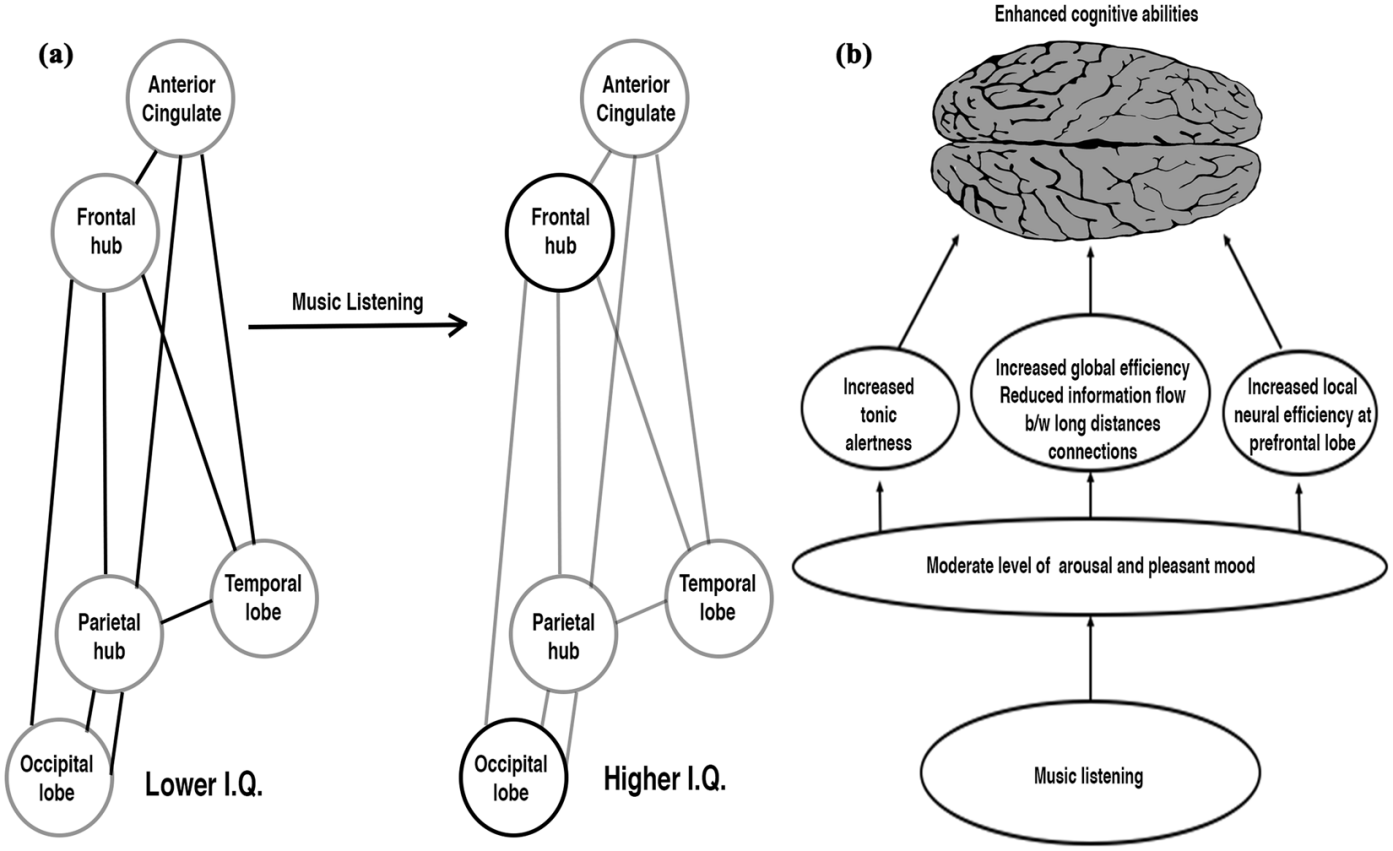
Schematic model (**a**) shows decreased information flow along distance connections in the P-FIT network coupled with increased efficiency at frontal and occipital lobes on music listening. Dark and grey lines indicate high and low information flow in the interconnections respectively while dark and grey circles represent high and low efficient hubs respectively, (**b**) Music with moderate level of arousal and pleasantness boost up the intelligence by (1) enhancing tonic alertness (occipital lobe), (2) increasing local processing in the region responsible for cognitive performance (prefrontal lobe), and (3) increasing global efficiency through purging of neural noises.

Earlier studies have shown that long-distance inter-electrode connections of the brain are more significantly correlated than the short ones^28,44^, and so also information flow in the left hemisphere of the brain had been reduced more than the right hemisphere in the individuals with high IQ^28,45^. We obtained more significant correlations for long-distance inter-electrode connections, and prominently in the left hemisphere in all the three analyses. Furthermore, findings show connections with inter-electrode distance of 12 cm or more were the most optimized, with many of them present between frontal and parietal lobes in all three analyses. This supports the small world model of intelligence by attenuating long-distance inter-electrode connections between the two hubs - frontal and parietal. In addition to the frontal-parietal hub interconnections, PSI analysis showed reduced long-distance communication at frontal-central, frontal-temporal, and intra-frontal regions. Also, phase coherence and phase delay analyses showed reduced communication at frontal-occipital, parietal-central, parietal-temporal, and parietal-occipital regions after the music stimulus for long-distances inter-electrode connections. Both results are in line with earlier studies^24,28^ that had demonstrated that long-distance communication in frontal-occipital, frontal-temporal, frontal-central, intra-frontal, parietal-occipital, and intra-parietal decreased significantly in intelligent individuals. This could be interpreted as listening to music also reduces the demands along other networks associated with intelligence such as occipital, temporal, cingulate cortex, and association cortex between frontal and parietal lobe in addition to the prominent role of frontal-parietal hub (Fig. 5a). This is in line with the recent development in the intelligence model^30,31^. Curtailing down the irrelevant connections of brain networks is vital to conserve energy for focused, undistracted, efficient functioning. Hence, music, by subsiding unrelated long-distance connections, enhances global efficiency, thereby boosting cognitive abilities. These results are well in accordance with the NEH theory, and previous studies^24,28^.

The regions specified by the phase analyses were slightly different. Phase coherence and phase delay analyses essentially pointed to the same areas of the brain (prominently at the parietal/occipital-central and parietal/occipital-frontal regions). This may be because both are power independent phase analyses. The results obtained through PSI analysis (prominently at the frontal-central, intra-frontal and frontal-parietal regions) were different from that of phase coherence and phase delay analyses. PSI analysis incorporates power information of the regions while calculating information flow among the respective regions. So, results from PSI analysis are likely to be affected by power fluctuation. For example, post music, there is a significant increment in power at the occipital lobes (O1, O2, Oz), prefrontal lobe (Fp2) and a feeble increment (although not significant) at parietal lobe (Fig. 4a). This leads to the absence of these regions in PSI analysis.

Alpha power is inversely related to brain activation. High power endorses diminished brain activation implying a more efficient brain. Hence, we next probed changes in the alpha power of the brain, particularly at the prefrontal and the occipital cortex. Power analysis shows an increase in the alpha power at all the three sites of occipital cortex and at the frontal cortex, particularly at the prefrontal site (Fp2) on exposure to music (Fig. 4a). The enhancement of the alpha power at the occipital lobe was more pronounced and stable than at the prefrontal lobe, which became significant only for a brief period. Enhanced alpha power at the occipital and frontal sites have been shown to be biomarkers of individuals with high IQ in the earlier studies^24,26^. Heightened alpha power at prefrontal lobe points towards increased neural efficiency in the prefrontal lobe, which is a site consistently linked to all three different types of functioning intelligence (spatial, verbal and circles)^46^. The findings are well in accordance with the NEH theory. Moreover, increased alpha oscillation power at the occipital site is also connected to increased tonic alertness or vigilance^47,48^ and internally directed attention^49^. This enhanced alertness may also trigger a temporary enhancement of cognitive functions^35–37^. Thus, music listening boosts-up cognitive abilities directly by increasing the local neural efficiency, and indirectly by enhancing sustained attention.

The results show enhancement in cognitive performance as a direct consequence of listening to music. The data also shows that irrespective of its type - Western/Mozart, as repeated in previous studies, or Indian classical music, as used in the present study - music enhances cognitive performance, thus proving EEG signals as a biomarker. Furthermore, the piece of music stimulus chosen for the study was able to induce, in the participants a significantly moderate level of pleasantness, which is an important prerequisite reported in many earlier studies^11^. The enhancement of cognitive functioning on exposure to music is not an artifact of arousal and mood as is sometimes understood. Our results show that a pleasant music with a moderate arousal level can actually trigger neural mechanism for boosting intelligence.

In summary, we propose that music listening affects the brain dynamics by three possible mechanisms (Fig. 5b) —

1. by increasing global efficiency through purging off irrelevant neural networks, especially between long-distance inter-electrode connections,
2. by increasing local neural efficiency at the prefrontal lobe,
3. by enhancing sustained attention.

While all the three mechanisms are independent, they reinforce each other, such as purging off irrelevant neural networks on listening to music rewards in two ways— (1) conserves brain’s vital energy which can be reallocated for enhanced local processing at the prefrontal lobes. (2) enhances sustained attention by reducing distraction from unrelated cross talks among neural networks. Sustained attention on the other hand also resists distraction thereby reduces unnecessary cross talks and boost up focussed functioning thereby reinforcing prefrontal lobe processing^32^. Similarly, efficient focussed functioning at prefrontal lobe favors sustained attention and less distraction. In the hierarchical framework of stimulus-mechanism-end effects, these three mechanisms provide an intermediate bridge to transform the arousal-mood status into the enhanced cognitive performance output level from the brain.

Our novel model gives clear possible neural mechanisms responsible for enhanced cognitive effect on exposure to music. It not only integrates the earlier two hypotheses of a) arousal and mood, and b) direct influence, but also reveal the detailed direct neural mechanisms induced by music on brain cognitive functions. However, the current model needs to be tested with other music especially Mozart’s which had earlier shown the enhanced cognitive effect. We have also interpreted the reduction in communication between the regions connected with intelligence such as occipital, temporal, cingulate cortex, and association cortex between frontal and parietal lobe as caused by music stimulus for enhancing cognitive performances. Further studies need to be done to verify whether there is a causal connection between them or the reduction in the information flow is caused through some other unrelated mechanisms. An interesting extension of the present study could be to compare the characteristics of the music stimulus used in our experiment with that of scale free brain music obtained from sonification of the post music EEG data^20,21^.

## Methods

### Participants

20 undergraduates of a technology institute participated in the study. The age of the participants ranged from 21 to 29 years (mean age = 24.06 years, SD = 2.69). No formal or informal training in music and right handedness were the inclusion criteria whereas hearing disorder, neurological disease, and usage of psychoactive drugs in the recent time were the exclusion criteria. We included only the male participants since the biomarkers for intelligence network are shown to have some differences between a male and a female^22^. The study was approved by the Institutional Ethics Committee (IEC) for research involving human subjects of the Indian Institute of Technology, Kanpur (IEC Communication no: IITK/IEC/2017-18 I/3). All the experiments were performed in accordance with relevant guidelines and regulations. The participants were recruited through in-house advertisement. Those who volunteered for the study were briefed about the experimental protocol. The experiment was conducted after filling the informed consent form.

### Stimulus and Experimental Procedure

Raga Darbari, one of the most popular ragas in Indian classical music system, is known to be effective in managing type 2 Diabetes^50^, insomnia^51^ and stress-related disorder^52–54^. Given the literary evidence from earlier studies^50–54^ involving Raga Darbari, we decided to choose Raga Darbari as the experimental stimulus. The socio-cultural milieu in India gives an inadvertent exposure to Ragas and Gharanas (schools) to all who are born and brought-up in the Indian society. Individuals coming from this background might not have received detailed and explicit knowledge of Ragas but have a subconscious familiarity with them. The stimulus used in the current experiment has cultural salience. A Raga composition features two parts namely alaap followed by gat. Alaap is note by note presentation of the raga, characterized by slow tempo without any rhythmic cycle. Gat section is characterized by fast tempo with rhythmic cycle provided by a percussion instrument. The length of our stimulus was 9 min 53 seconds. The alaap section was 3 min 22 seconds in length and the gat section was 6 min 31 seconds. The experimental session consisted of three conditions— pre-music silence condition for 275 seconds, music condition (listening to a segment of *Raga Darbari*), and post-music silence condition for 275 seconds. The experiment was conducted in a soundproof laboratory with the stereo speakers kept around 2 meters symmetrically from the participants. The lightening condition inside the room was dim. The participants were made to sit comfortably. They were instructed to listen to the audio stimulus attentively with their eyes closed during the whole experiment. A subjective assessment of their mood was also taken before and after completion of the experiment.

### EEG recording and preprocessing

EEG was recorded using a g.HIamp bio-signal amplifier (Guger Technologies, OG, Graz, Austria) at 32 scalp positions according to the international 10–20 system. The sampling frequency of 512 Hz and impedance level below 5 Kohms was kept. A band pass filter of 0.01–100 Hz was applied. Right earlobe was used as a reference electrode. To keep the recording free from eye artifacts, two electrodes, one above and one below the right eye and two electrodes near each eye’s outer canthus were also placed. The Data was high-pass filtered at 0.5 Hz to remove any DC drift and was visually checked for any contamination due to eye movement, muscle movement, or electrodes movement. Bad electrodes were marked if any. Independent component analysis (Infomax ICA algorithm, runica) was applied on the good channels to remove further artifacts. Bad channels were then interpolated (Spherical interpolation). The first 275 seconds’ pre-music silence condition provided the baseline whereas the 275 seconds’ post-music silence condition was helpful to study the fading effect of music. The data of three participants were removed from the analyses due to heavy artifacts whereas two participants who had previous exposure to music were also excluded from the study.

### Phase and Power analyses

The effect of music is for a short duration. Pre-music and post-music silence conditions were juxtaposed in various time periods to find the period of maximum efficiency. Phase and power analyses were performed as a function of time period rather than time in order to increase the signal to noise ratio. The time periods selected for analyses were 25 seconds, 50 seconds (2^nd^ time period) till 275 seconds (11^th^ time period). We digitally filtered the EEG data for 8–13 Hz range to obtain alpha band signals. We applied Hilbert transform to the filtered data to obtain instantaneous phase and power of the EEG signal. We calculated phase coherence, phase delay and phase slope index for all the time periods for both pre-music and post music silence conditions. We selected the time period for which the phase analyses results showed maximum reduction in the information flow by listening to music. We investigated the alteration in the topography of functional connectivity through phase coherence, phase delay and PSI analyses for the corresponding time period between Pre-music and post-music silence conditions. We also studied variation in the alpha power at the prefrontal and occipital lobes of the brain for the corresponding time period.

### Statistical analysis

EEG data recorded at the scalp positions may not necessarily be Gaussian, and in addition, our sample size was not sufficient for the validity of the parametric test, so we applied a non-parametric test in our EEG analysis. Wilkinson sign rank test was applied for statistical testing for power and phase analyses. We used t-test for comparing mean values, subjective assessment of music listening and brain lateralization analysis. To investigate information flow variation with respect to inter-electrode distances in the brain network, a repeated ANOVA was applied. All the statistical comparisons were two-tailed with the *α-*value set to 0.05.

### Hilbert Transform

EEG time series is time-varying and has only real component. For the purpose of obtaining instantaneous power as well as phase, we need a complex value signal. Hilbert transform function can extract the imaginary part from a real value signal and forms a complex signal called Analytical signal. Let *R*_*r*_(*t*) represent a real signal. Then the Hilbert transform of the *R*_*r*_(*t*) signal is obtained by taking convolution of *R*_*r*_(*t*) with the function *h*(*t*) = 1/(π*t*).

1 $${R}_{at}(t)={R}_{r}(t)+\iota {R}_{ht}(t)$$

2 $${R}_{ht}(t)=\frac{1}{\pi }P.\,V{\int }_{-\infty }^{+\infty }\,\frac{{R}_{r}(\tau )}{{R}_{r}(t-\tau )}d\tau $$

where *R*_*at*_(*t*) represents the analytical signal, and *R*_*ht*_(*t*) represents the Hilbert transform of the real signal. Since the function *h*(*t*) is non-integrable, the Hilbert transform is defined using Cauchy principal value (P.V). This gives the analytical signal *R*_*at*_(*t*), which can also be represented in polar form as

3 $${R}_{at}(t)=M(t)\ast {e}^{i\varphi (t)}\,$$

4 $$M(t)=\,\sqrt{{R}_{r}{(t)}^{2}+{R}_{ht}{(t)}^{2}}$$

5 $$\varphi (t)=arctan(\frac{{R}_{ht}(t)}{{R}_{r}(t)})$$

where *M*(*t*) denotes the instantaneous amplitude while *ϕ*(*t*) denotes instantaneous phase.

### Phase coherence

It is precisely defined as clustering of phase angle difference between two electrodes in polar space. Let *Z*_*i*_ and *Z*_*j*_ be two time-series for two channels *i* and *j* respectively. So, the instantaneous phase difference between them is:

6 $${\varphi }_{ij}(t)={\varphi }_{i}(t)-{\varphi }_{j}(t)$$

where *ϕ*_*i*_(*t*), and *ϕ*_*j*_(*t*) are instantaneous phase angle of the two-time series and *ϕ*_*ij*_(*t*) is instantaneous phase difference between them. Phase coherence is calculated as:

7 $$PC(f)=|\frac{1}{t}\,{\int }_{0}^{t}\,{e}^{i{\varphi }_{ij}(t)}dt|$$

where *PC*(*f*) represents phase coherence at the frequency *f*, *t* denotes calculation over time and *ϕ*_*ij*_(*t*) represents the instantaneous phase difference between the electrodes *i* and *j*^55^. Phase coherence is positively correlated with information flow.

### Phase delay

Phase delay is positively related to IQ and is found to be a robust biomarker for high IQ individuals^24^. Phase delay is defined as the absolute instantaneous phase difference between the electrodes as calculated in the phase coherence analysis. Its absolute value is computed by squaring the instantaneous phase difference and then taking its square root.

8 $$|{\varphi }_{ij}(t)|=\,\sqrt{{\varphi }_{ij}{(t)}^{2}}$$

### Phase slope index (PSI)

Phase Slope Index (PSI) is positively correlated with information flow and thus inversely related to IQ^28^. The basic principle for PSI is that a temporal order between two-time series gives rise to a phase difference as a linear function of frequency^56^. For each frequency bin, PSI measures the change in the phase difference with its neighbourhood weighted by coherence. If for a particular band, there is significant spectral coherency and the phase difference changes consistently across the frequency bins, then the PSI value would drift from zero. PSI is calculated in the following way for the two time-series *Z*_*i*_ and *Z*_*j*_. The whole time-series is divided into *k* segments of *T* duration each. Cross and auto spectral density among them is defined as follows:

9 $${S}_{i,j}(f)=\frac{1}{k}\sum _{k}\,{Z}_{i}(f,k)\ast {Z}_{j}^{\ast }(f,k)$$

10 $${S}_{i,i}(f)=\frac{1}{k}\sum _{k}\,{Z}_{i}(f,k)\ast {Z}_{i}^{\ast }(f,k)$$

where *S*_*i*,*j*_(*f*) represents cross spectral density at the frequency *f*, *S*_*i*,*i*_(*f*) represents the auto spectral density at the frequency *f*, and *Z*_*i*_(*f*, *k*) represents the Fourier transform in channel *i* and segment *k*.

11 $${C}_{i,j}(f)=\frac{{S}_{i,j}(f)}{\sqrt{{S}_{i,i}(f)\ast {S}_{j,j}(f)}}$$

where *C*_*i*,*j*_(*f*) represents complex coherency. Calculation of PSI value is done as:

12 $${\psi }_{i,j}={\mathfrak{J}}\{\sum _{f\in F}{C}_{i,j}^{\ast }(f){C}_{i,j}(f+\delta f)\}$$

where *ψ*_*i*,*j*_ represents Phase slope index, *δf* represents frequency resolution, *F* represents the frequency band, and ${\mathfrak{J}}$ represents imaginary part of the complex coherency.

For further depth of the PSI, please refer Witham *et al*.^57^ and Vinck *et al*.^58^.

The inter-electrode distance between each pair of electrodes was calculated as Euclidian distance between them based upon the positioning of the electrodes according to the international 10–20 system. Long-distance connection between Fp1-O1 electrode pairs was 24 cm as used by Thatcher *et al*.^24,28^.

### Power analysis

Instantaneous power is calculated by taking square of the instantaneous amplitude.

13 $$A(t)=M{(t)}^{2}$$

14 $$A(t)={R}_{r}{(t)}^{2}+{R}_{ht}{(t)}^{2}$$

### Subjective Assessment of Music Listening

An assessment of participants’ moods was taken before and after the experiment. They were asked to rate their experience between pleasant and unpleasant on an 11-point Likert scale.

## Electronic supplementary material

Supplementary information

## Data Availability

The data used in the current investigation are accessible upon reasonable request from the corresponding author. All the codes were written on MATLAB platform and open source toolboxes such as EEGLAB and Fieldtrip were also used.
