# Supplementary material for: Short-term enhancement of cognitive functions and music: A three-channel model
Source: Sci Rep. 2018 Oct 19;8:15528. doi: 10.1038/s41598-018-33618-1 (PMC6195580; doi:10.1038/s41598-018-33618-1)
Supplement: Supplementary file 1 — Supplementary information [file 41598_2018_33618_MOESM1_ESM.docx]

**Supplementary material:**

**Short-term enhancement of cognitive functions and music:**

**A three-channel model**

**Ashish gupta^1^, Braj Bhushan^2^, and Laxmidhar Behera^1,*^**

**Indian Institute of Technology, Department of Electrical Engineering, Kanpur, 208016, India.**

**^2^Indian Institute of Technology, Department of Humanities and Social Sciences, Kanpur, 208016, India.**

***Correspondence and requests for materials should be addressed to L.D. (lbehera@iitk.ac.in).**

**Supplementary section S1: - Schematic model of Phase analysis results:**

Figure S1 depicts the functional connectivity of the brain as obtained after phase analysis. Phase coherence analysis shows reduced information flow after listening to music. The inter-connections with reduced communication were located between parietal/occipital brain regions with frontal, central, and temporal brain regions as well as between parietal and occipital brain regions (fig. S1a). Phase delay analysis also shows reduced information flow post exposure to music. The inter-connections with reduced communication were located between parietal/occipital brain regions with frontal, central, and temporal brain regions as well as between parietal and occipital brain regions (fig. S1b). Phase slope index analysis shows connections, located between frontal region with central, parietal, and temporal regions as well as located at intra-frontal region, to have significantly reduced PSI value after listening to music (fig. S1c).


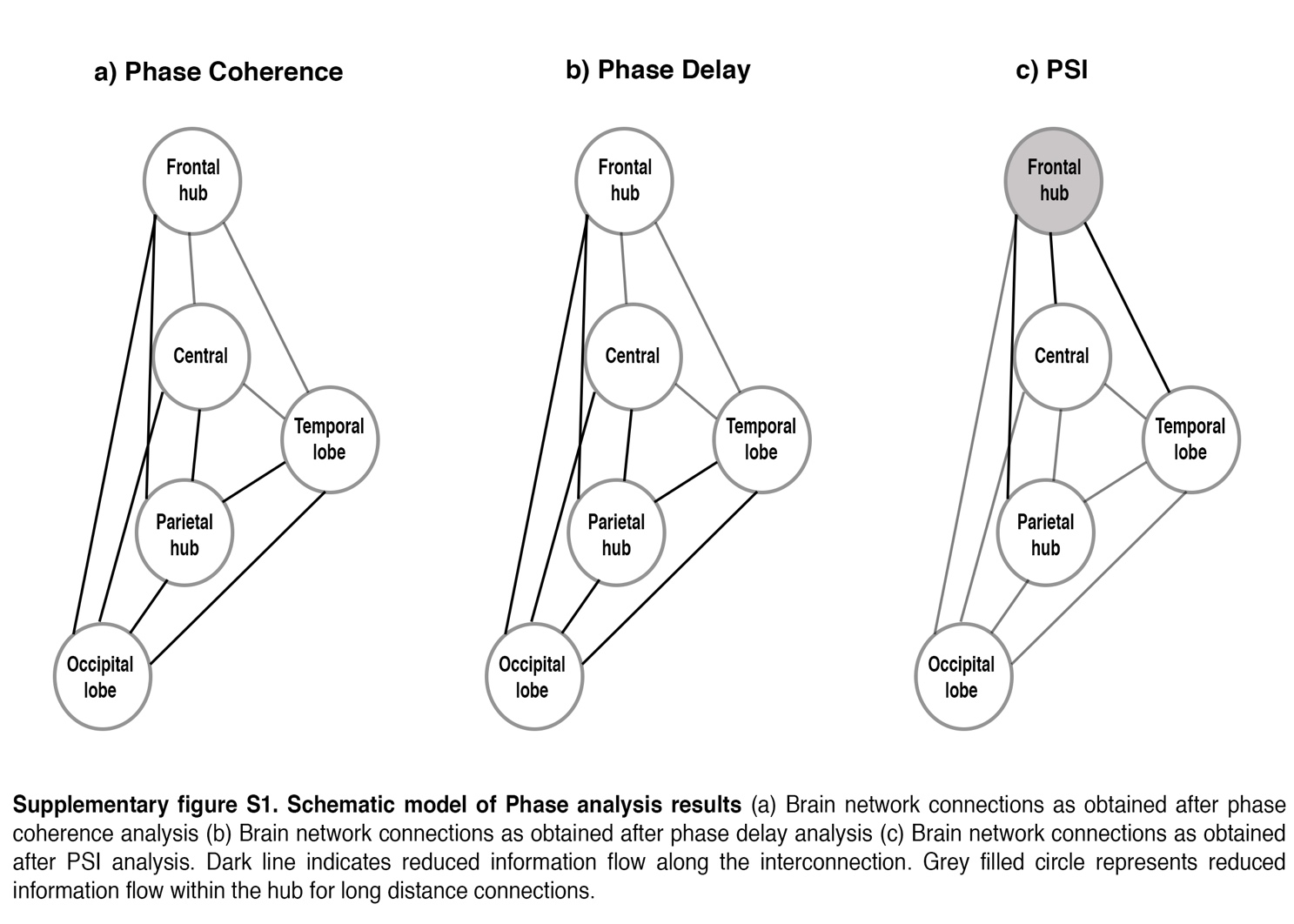


**Supplementary section S2: - Spectral Coherence analysis:**

We analysed the brain state for the duration of 100 seconds based on spectral coherence analysis also and observed a significant reduction post music. Figure S2(a) shows the brain network inter-connections (spectral coherence variables) which showed a significant reduction (Wilkinson sign rank test, p < .05) in spectral coherence on exposure to music with Z value ranging between 1.9879 to 2.5558 and effect size between 0.3629 to 0.4666. Most of the connections (27 in total) were located between parietal/occipital regions with frontal and central regions while a few were with temporal brain regions. Figure S2(b) shows a comparison between the mean values of the 27 connections. It shows a significant reduction after music stimulus (t = 4.4587, df = 14, p < .001, effect size = 1.1512). Figure S2(c) shows the number of connections with reduced spectral coherence as a function of inter-electrode distances. A repeated measure ANOVA with a Greenhouse-Geisser correction indicates a significant effect of inter-electrode distances on the number of connections (F_1.340,18.759_ = 153.093, p < .001). Post hoc comparisons using Bonferroni correction show that connections between 6-12 cm (Mean = 5.4, SD = 1.844) were greater in number than that of less than 6 cm (Mean = 1.4, SD = .632) and the difference was statistically significant (p < .001) while connections more than 12 cm (Mean = 13.87, SD = 3.603) were significantly greater than that of 6-12 cm (p < .001) and also greater than that of less than 6 cm (p < .001). Thus, long distances connections were affected more than the short distances ones. Figure S2(d) shows that the left hemisphere of the brain was more affected and correlated as compared to the right hemisphere (t = -3.3038, df = 14, p< .05, effect size = -0.8530). The results show reduced communication post music and is in line with that obtained through phase coherence analysis.

**
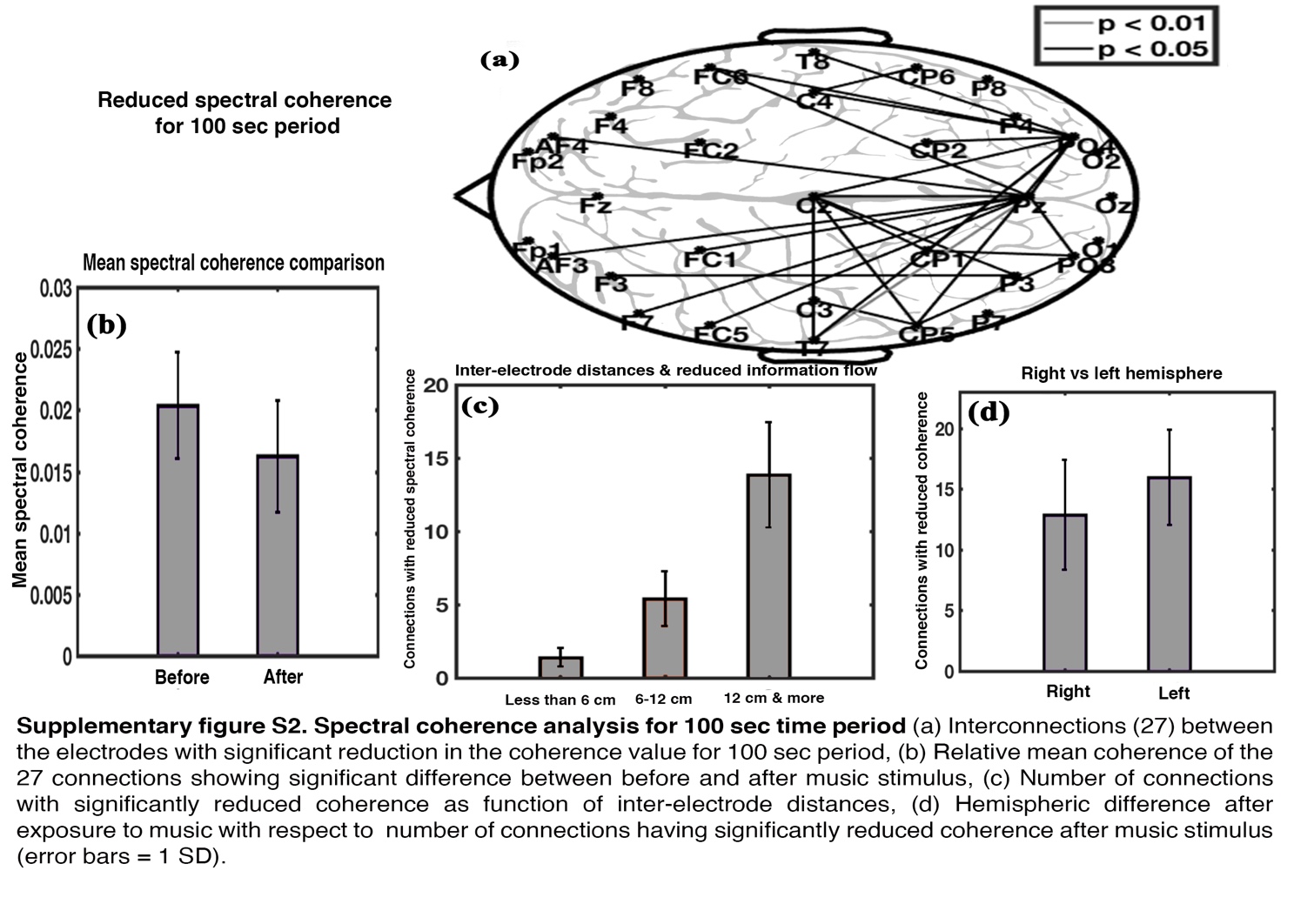
**

**Supplementary section S3: - Power analysis as function of time:**

Figure S3(a) shows power analysis as a function of time period. Power analysis as function of time is shown in Fig. S3(b). We analysed a period of 25 seconds that is 0-25 seconds, 25-50 seconds, 50-75 seconds and so on for pre and post music silence condition. Reconfirming the previous analysis, occipital cortex has been found to have significant enhancement (p < .05) in power post music at all the three electrode locations (O1, O2, Oz) along with parietal-occipital cortex (PO3). Electrode O1 (Z = -2.9534, p < .01, effect size = -0.5392), O2 (Z = -2.3854, p < .05, effect size = -0.4355), and Oz (Z = -2.9534, p < .01, effect size = -0.5392) showed a maximum enhancement at the time between 75 to 100 seconds. PO3 electrode showed maximum increment in power between 200 to 225 seconds (Z = -2.5558, p < .05, effect size = -0.4666). Fp2 showed maximum significant enhancement between 150 to 175 seconds (Z = -2.7262, p < .01, effect size = -0.4977) while Fp1 showed maximum significant increment between 200 to 225 seconds (Z = -2.3854, p < .05, effect size = -0.4355). These results reconfirm the findings obtained in power analysis as a function of time period.


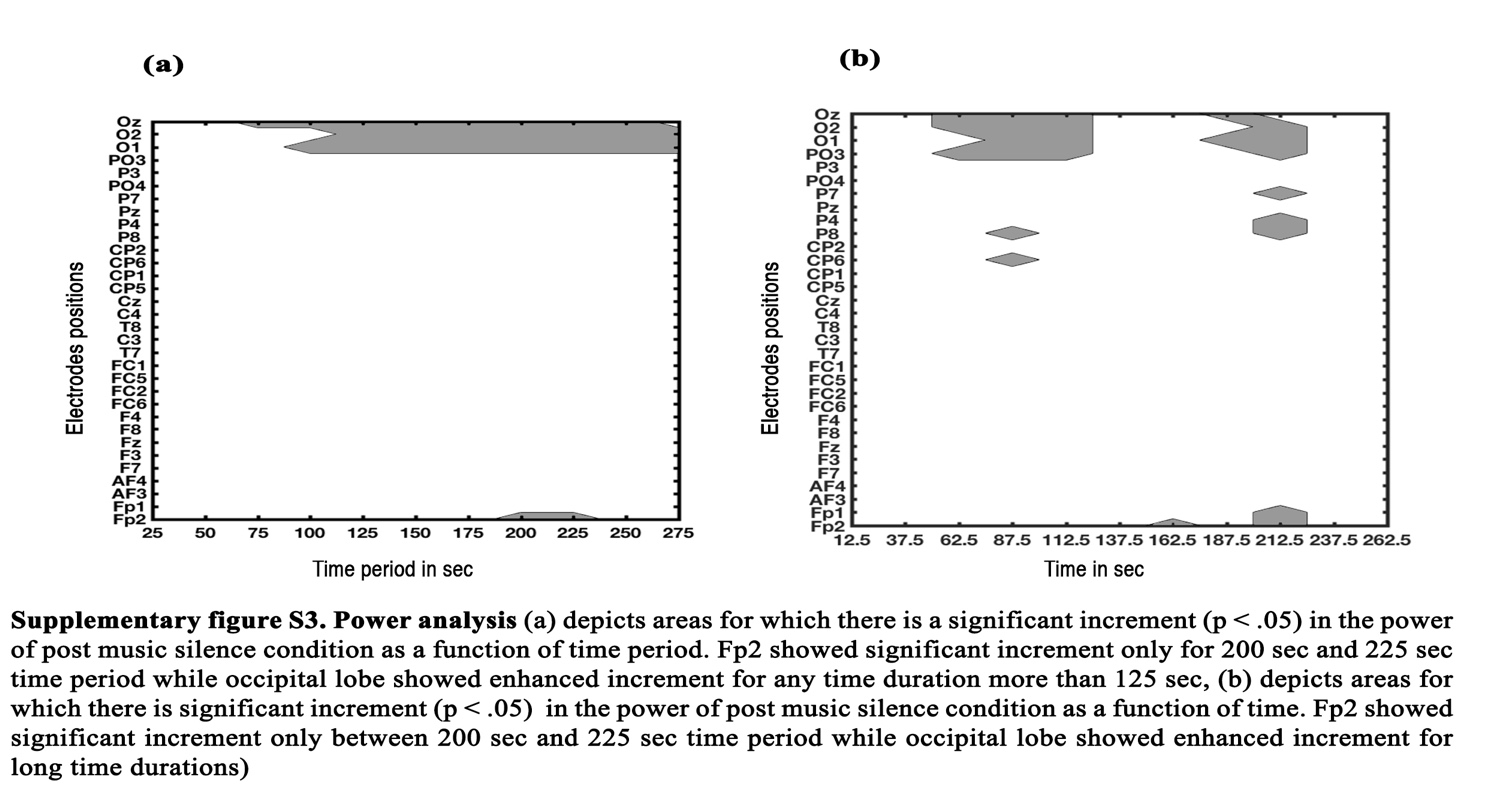


We ran our EEG data analysis with REST reference. The results obtained are as follows—

**Supplementary section S4: - Power analysis with REST referencing.**

**Results obtained through REST Referencing: -**

**Power analysis: -**

Earlier spectral power analysis has shown that there is a distinct difference in power values and topographical distribution of power for different reference choices^[[1]](#endnote-1),^^[[2]](#endnote-2)^. However, the primary power distribution areas are found to be consistent across reference choices^2^. We performed power analysis on our EEG data with REST reference. We examined alpha power variation in the brain resting state after listening to music. We examined the prefrontal and occipital lobes which are correlated with general intelligence^[[3]](#endnote-3)^^,^^[[4]](#endnote-4)^.


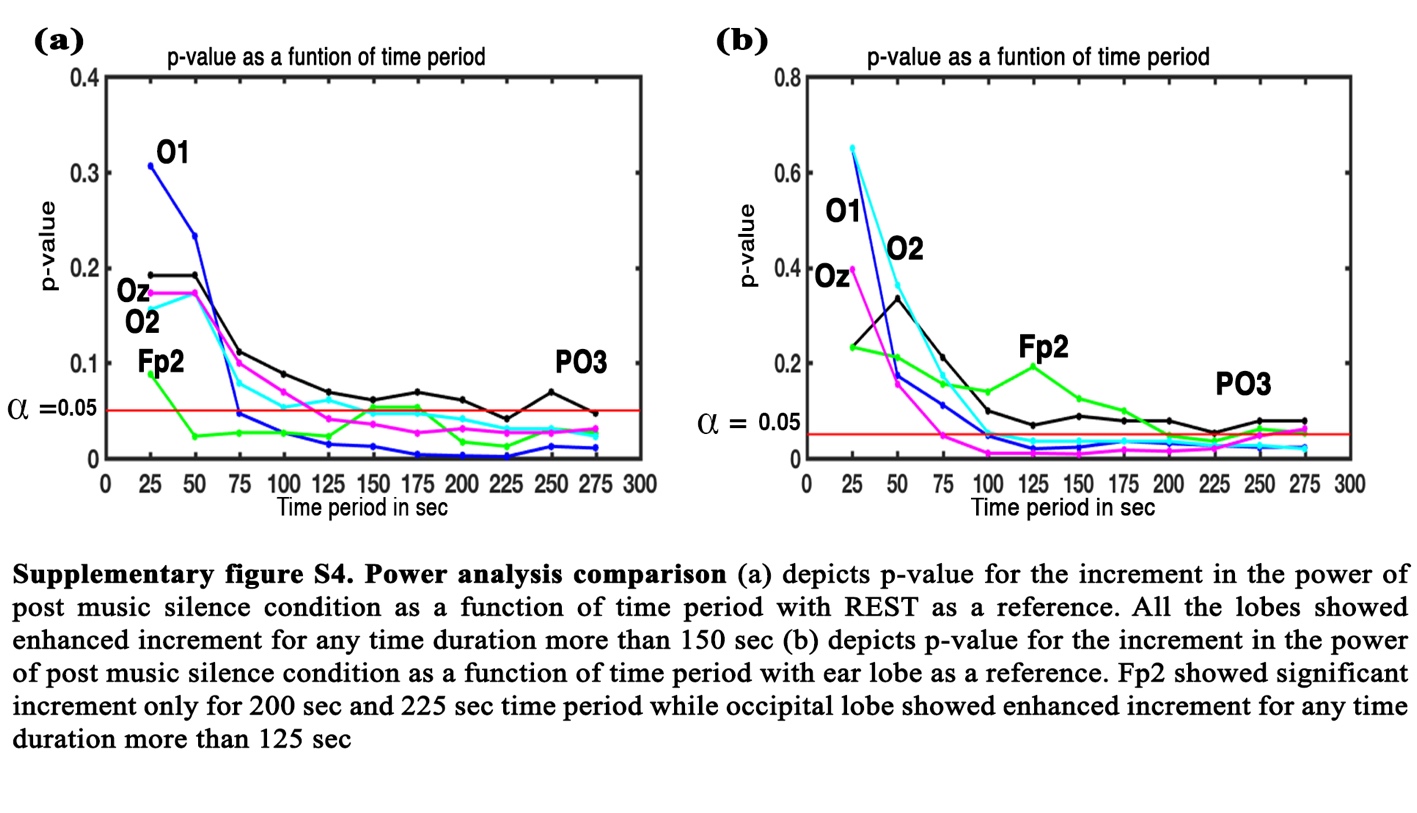


The results obtained through EEG analysis with REST as reference are consistent with that obtained with ear lobe as reference. We found significant increment of alpha power values post music silence condition as compared to pre-music silence condition at the prefrontal area (Fp2) and at all the three site of the occipital lobe (O1, O2, & Oz). All these sites have been linked with general intelligence^3,4^. All the three sites of occipital lobe showed significant enhanced power (signifying increased sustained attention in addition to intelligence) 150 seconds onwards (as also obtained in analysis with ear lobe as reference). Post 50 seconds, we obtained a significant increment of power at prefrontal area (Fp2) (signifying an enhanced prefrontal processing) as compared to little increment of short duration in EEG analysis with ear lobe as reference. Electrode Fp2 showed a maximum enhancement at the time period of 225 seconds (Z = - 2.4990, p < .05, effect size = -0.4563), O1 at 225 seconds’ period (Z = -3.1238, p < .05, effect size = -0.5703), O2 at 225 seconds’ period (Z = -2.1583, p < .05, effect size = - 0.3940), while Oz at also 225 seconds’ period (Z = -2.2151, p < .05, effect size= -0.4044). Thus, the results obtained through EEG power analysis with REST reference, show enhanced cognitive abilities. The results also support the findings obtained in the earlier EEG analysis with unilateral ear lobe as reference.

We did phase analysis with REST reference and the results are shown below. We examined the variations in the information flow between long-distance connections in alpha band.

**Phase analysis: -**

**Supplementary section S5: - Phase delay analysis with REST Referencing**

Phase delay analysis results: -


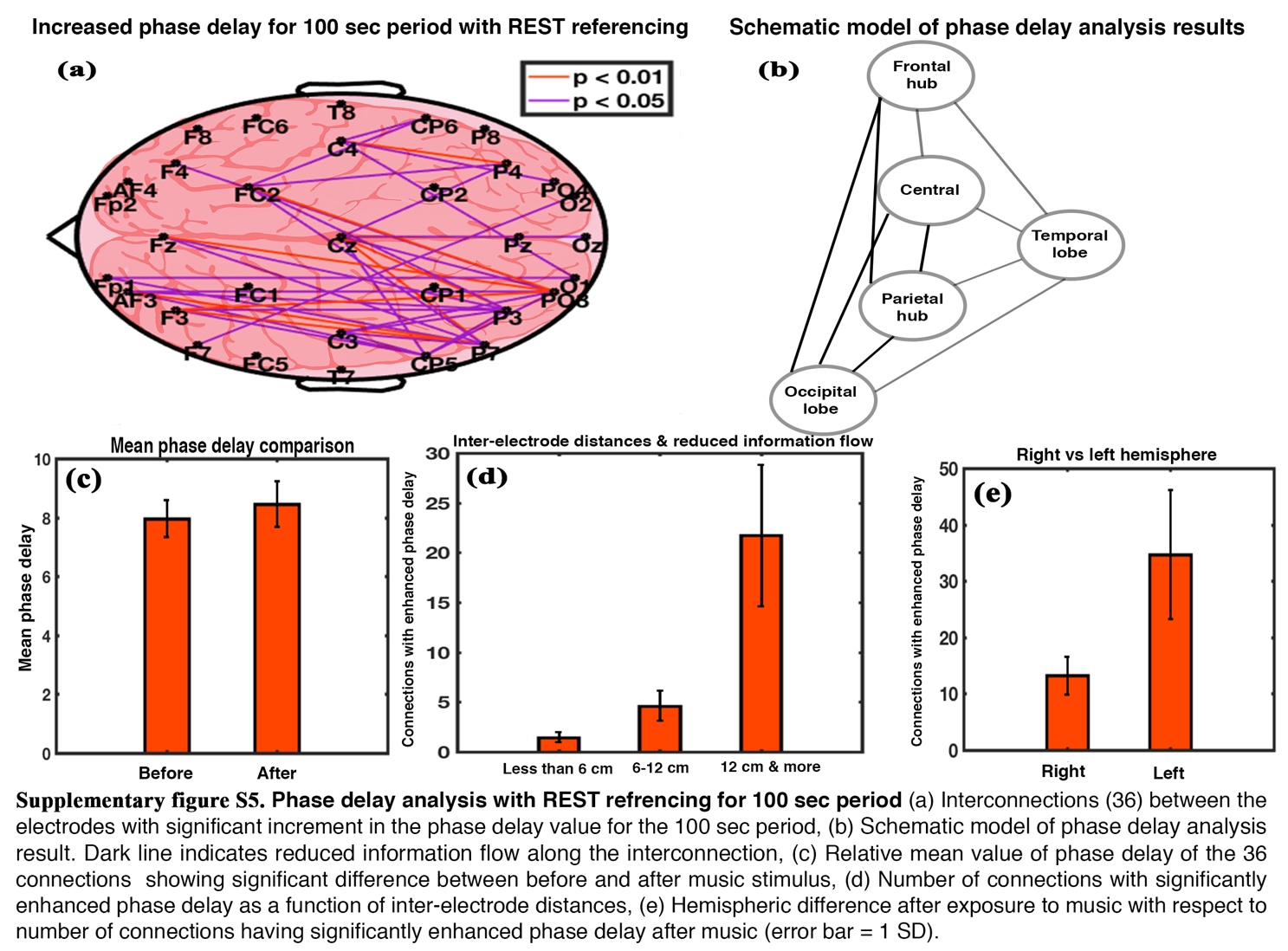


We analysed the brain state for the duration of 100 seconds through phase delay analysis with REST referencing. Figure S5(a) shows the brain network inter-connections with a significant increment (Wilkinson sign rank test, p < .05) in phase delay on exposure to music with Z value ranging between -1.9879 and -2.7830 and effect size between -0.3629 and -0.5081. We found the connections (36 in total) between parietal/occipital regions and frontal & central regions as well as between parietal and occipital brain regions. Figure S5(b) shows schematic model of phase delay analysis result. Dark line indicates reduced information flow along the interconnection. Figure S5(c) shows a comparison between the mean value of the 36 connections. It shows a significant increment after music stimulus (t = -4.3501, df = 14, p < .001, effect size = -1.1232), signifying a net enhancement in brain efficiency. Figure S5(d) shows the number of connections with enhanced phase delay as a function of inter-electrode distances. A repeated measure ANOVA with a Greenhouse-Geisser correction indicates a significant effect of inter-electrode distances on the number of connections (F_1.038,14.525_ = 119.169, p < .001). Post hoc comparisons using Bonferroni correction show that the connections in the range of 6-12 cm (Mean = 4.6000, SD = 1.5024) were greater in number than those in the range of less than 6 cm (Mean = 1.4667, SD = .5164) and the difference was statistically significant (p < .001) while connections in the range of more than 12 cm (Mean = 21.7333, SD = 7.1160) were significantly greater than those in the range of 6-12 cm (p < .001) and also greater than those in the range of less than 6 cm (p < .001). Thus, long distance connections were affected more than the short distance ones. With respect to the hemispheric differences, left hemisphere of the brain had more number of connections with enhanced phase delay (Fig. S5(e)) as compared to the right hemisphere (t = -8.5761, df = 14, p < .001, effect size = -2.2143). The results show reduced communication post music along long distance connections and support the findings obtained through phase delay analysis with ear lobe as reference.

**Supplementary section S6: - Phase slope index analysis with REST Referencing**

Phase slope index results: -


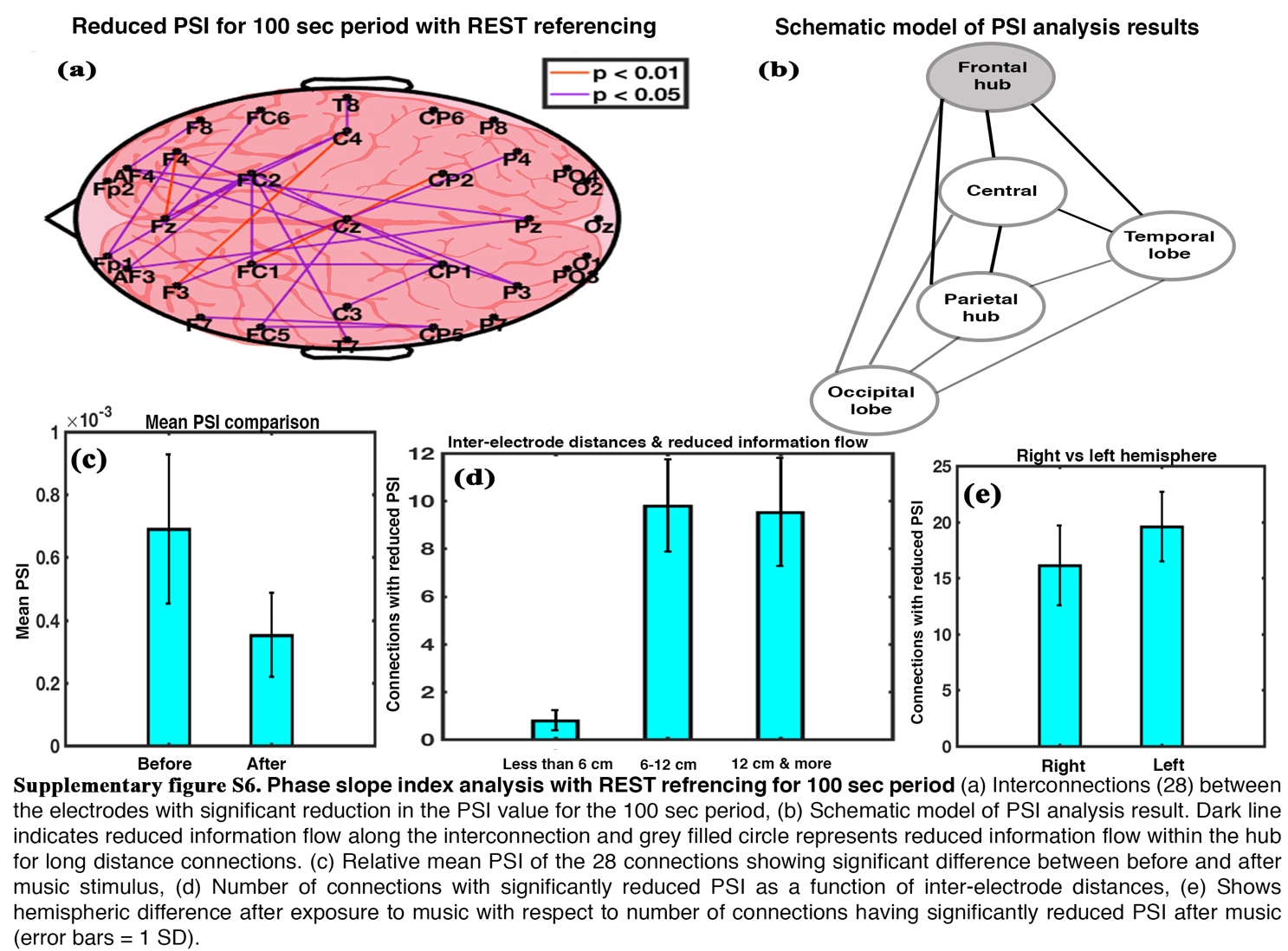


We analysed the brain state for the duration of 100 seconds through PSI analysis with REST referencing. Figure S6(a) shows the brain network inter-connections which showed a significant reduction (Wilkinson sign rank test, p < .05) in PSI measures on exposure to music with Z value ranging between 1.9879 and 2.7830 and effect size between 0.3629 and 0.5081. We found the connections (28 in total) between frontal regions and frontal, parietal, central & temporal regions as well as between central and temporal region. Figure S6(b) Schematic model of PSI analysis result. Dark line indicates reduced information flow along the inter-connections and grey filled circle represents reduced information flow within the hub for long distance connections Figure S6(c) shows a comparison between the mean value of the 28 connections. It depicts an effective reduction in brain network’s PSI (thus information flow) at 100 seconds, signifying a net enhancement in brain efficiency. We found a significant reduction after music stimulus (t = 4.6916, df = 14, p < .001, effect size = 1.2114). Figure S6(d) shows the number of connections with reduced PSI as a function of inter-electrode distances. A repeated measure ANOVA indicates a significant effect of inter-electrode distances on the number of connections (F_2,28_ = 168.696, p < .001). Post hoc comparisons using Bonferroni correction show that connections in the range of 6-12 cm (Mean = 9.8000, SD = 1.9346) and connections in the range of more than 12 cm (Mean = 9.5333, SD = 2.2636) were greater in number than those in the range of less than 6 cm (Mean = 0.8000, SD = 0.4140) and the difference was statistically significant (p < .001). There was no significant difference between the number of connections in the range of more than 12 cm (Mean = 9.5333, SD = 2.2636) and those in the range of 6-12 cm (p > .05). Thus, long distance connections were affected more than the short distance ones. With respect to the hemispheric differences, left hemisphere of the brain had more number of connections with reduced PSI (Fig. S6(e)) as compared to the right hemisphere (t = - 4.7526, df = 14, p< .001, effect size = - 1.2271). The results show reduced communication post music along long distance connections and support the findings obtained through phase slope index analysis with unilateral ear lobes as reference.

**Supplementary section S7: - Phase coherence analysis with REST Referencing and Discussion**

Phase coherence analysis results: -

We obtained a significant reduction in the phase coherence value along long-distance connections in the post music silence condition as compared to the pre-music silence condition with ear lobe as reference. In case of EEG data analysis with REST as reference we did not observe any such significant reduction in the phase coherence value except only in three interconnections (CP6-C4, CP5-P3, and CP5-PO3).

**Discussion: -**

The results obtained through power analysis with REST referencing support the findings obtained through the power analysis with right ear lobe as reference. They show enhanced cognitive processing post music. Particularly, power analysis results with REST referencing support the two mechanisms of our model— 1) increased local neural efficiency at the prefrontal lobe and, 2) enhanced sustained attention.

The results obtained through phase delay analysis and PSI analysis with REST as reference supports the findings obtained through phase analysis with ear lobes as reference. Results show significant reduction in the information flow after listening to music especially along the long-distance interconnections and in the left hemisphere. Particularly, it supports the following mechanisms of our model— 1) Increasing global efficiency through purging off irrelevant neural networks especially between long-distance inter-electrode connections. However, the results of phase coherence analysis with REST referencing are not consistent with the findings obtained in phase coherence analysis with ear lobes as reference.

Limitation of referencing in comparative study: -

The choice of reference plays a very important role in studying the brain functional connectivity and in comparing the results obtained from various labs with different references^1,2^. The choice of reference also affect the graph network properties such as node degree and local efficiency^[[5]](#endnote-5)^. Recent simulation studies have shown that the REST referencing is able to perform best under varied situations^1,2,5^. However, there are significant differences in the network topography among the various choices of references^1,2,5^. For example, study conducted by Qin *et. al.*^2^ have found that REST and common average referencing are characterized with the connections between the anterior and the posterior part of the brain in the alpha band (based on phase coherence measure) although strength of the connections are different in both the cases. EEG phase coherence analysis, when done with mastoid (linked as well as unilateral) on the same EEG data, shows no such connections between anterior and posterior part of the brain^2^. Other simulation study has shown that functional network topography between REST referencing and common average referencing (based on phase coherence measure) also differ^[[6]](#endnote-6)^. Similar results are obtained in another study^[[7]](#endnote-7)^, suggesting that changing the reference lead to alteration in the network topography as well as individual strength of the connections. Indeed, performance of references depend upon the nature of cognitive task performed^[[8]](#endnote-8)^, orientation of the dipole sources^6^, signal to noise ratio^6,7^, and frequency band under investigation^2^.

Hence, differences in measuring the significant changes between pre-treatment vs post treatment group in already distinct networks under distinct references is possible. This would partially explain the difference observed in the result in the phase coherence analysis. However, a detailed understanding of the effect of reference on the network topographical characteristics between pre-treatment vs post treatment in group analysis is not well established and is a scope for future research.

Since brain functional connectivity is dependent upon the choice of network^1,2,6,7^ so comparing the results across different referencing scheme become a problem. More work on the comparative study of the brain functional connectivity with varied referencing scheme, cognitive tasks and for group analysis (pre-treatment vs post treatment) is needed. REST referencing is, however, a most suitable method to recover the true functional connectivity of the brain networks under varied circumstances^1,2,6,7^. Hence, contrasting and comparing the brain network connections under different reference scheme with that with under REST referencing can provide a solution to this issue. We have accordingly provided the results with REST referencing in the supplementary section.

Overall, the results obtained through power and phase analysis with REST referencing support the findings obtained with right ear lobe as reference.

1. **.** Yao, D. et al. A comparative study of different references for EEG spectral mapping: the issue of the neutral reference and the use of the infinity reference. *Physiological measurement*, **26(3),** 173 (2005). [↑](#endnote-ref-1)
2. . Qin, Y., Xu, P., & Yao, D. A comparative study of different references for EEG default mode network: the use of the infinity reference. *Clinical neurophysiology*, **121(12),** 1981-1991 (2010). [↑](#endnote-ref-2)
3. **.** Thatcher, R. W., North, D. & Biver, C. Eeg and intelligence: relations between eeg coherence, eeg phase delay and power. *Clin. neurophysiology* **116,** 2129-2141 (2005). [↑](#endnote-ref-3)
4. **.** Marosi, E. et al. Broad band spectral eeg parameters correlated with different iq measurements. *Int. J. Neurosci.* **97,** 17-27 (1999). [↑](#endnote-ref-4)
5. **.** Chella, F., Pizzella, V., Zappasodi, F., & Marzetti, L. Impact of the reference choice on scalp EEG connectivity estimation. *Journal of neural engineering*, **13(3),** 036016 (2016). [↑](#endnote-ref-5)
6. **.** Huang, Y. et al. How different EEG references influence sensor level functional connectivity graphs. *Frontiers in neuroscience*, **11,** 368 (2017). [↑](#endnote-ref-6)
7. **.** Chella, F., Pizzella, V., Zappasodi, F., & Marzetti, L. Impact of the reference choice on scalp EEG connectivity estimation. *Journal of neural engineering*, **13(3),** 036016 (2016). [↑](#endnote-ref-7)
8. **.** Rappelsberger, P. The reference problem and mapping of coherence: a simulation study. *Brain topography*, **2(1-2),** 63-72 (1989). [↑](#endnote-ref-8)
